# Drug Repositioning by Kernel-Based Integration of Molecular Structure, Molecular Activity, and Phenotype Data

**DOI:** 10.1371/journal.pone.0078518

**Published:** 2013-11-11

**Authors:** Yongcui Wang, Shilong Chen, Naiyang Deng, Yong Wang

**Affiliations:** 1 Key Laboratory of Adaptation and Evolution of Plateau Biota, Northwest Institute of Plateau Biology, Chinese Academy of Sciences, Xining, China; 2 College of Science, China Agricultural University, Beijing, China; 3 National Center for Mathematics and Interdisciplinary Sciences, Academy of Mathematics and Systems Science, Chinese Academy of Sciences, Beijing, China; 4 Molecular Profiling Research Center for Drug Discovery, National Institute of Advanced Industrial Science and Technology, Tokyo, Japan; Chinese Academy of Sciences, China

## Abstract

Computational inference of novel therapeutic values for existing drugs, i.e., drug repositioning, offers the great prospect for faster and low-risk drug development. Previous researches have indicated that chemical structures, target proteins, and side-effects could provide rich information in drug similarity assessment and further disease similarity. However, each single data source is important in its own way and data integration holds the great promise to reposition drug more accurately. Here, we propose a new method for drug repositioning, PreDR (**Pre**dict **D**rug **R**epositioning), to integrate molecular structure, molecular activity, and phenotype data. Specifically, we characterize drug by profiling in chemical structure, target protein, and side-effects space, and define a kernel function to correlate drugs with diseases. Then we train a support vector machine (SVM) to computationally predict novel drug-disease interactions. PreDR is validated on a well-established drug-disease network with 1,933 interactions among 593 drugs and 313 diseases. By cross-validation, we find that chemical structure, drug target, and side-effects information are all predictive for drug-disease relationships. More experimentally observed drug-disease interactions can be revealed by integrating these three data sources. Comparison with existing methods demonstrates that PreDR is competitive both in accuracy and coverage. Follow-up database search and pathway analysis indicate that our new predictions are worthy of further experimental validation. Particularly several novel predictions are supported by clinical trials databases and this shows the significant prospects of PreDR in future drug treatment. In conclusion, our new method, PreDR, can serve as a useful tool in drug discovery to efficiently identify novel drug-disease interactions. In addition, our heterogeneous data integration framework can be applied to other problems.

## Introduction

Drug repositioning is known as the ‘old drug, new disease’ paradigm. It aims to find new diseases to cure for existing drugs and thus offers the possibility for faster, safer, and cheaper drug development. Given the huge search space and the rapid accumulation of drug related data at molecular level, computational approaches are highly desired to narrow down the gap between medical indications and elucidation of drug effects [1]. In addition to their low cost and time-efficiency predictions, computational methods have the advantage in understanding the mechanisms of drug actions.

Drug takes effect via its protein targets in cell to cure disease. Thus, many previous studies in computational drug repositioning focused on the drugs with known downstream target proteins in disease-specific molecular networks [2]–[4]. However, low-throughput data limits the applications in small scale. Recent accumulated high-throughput data for both drugs and diseases provide possibilities to uncover novel statistical associations between drugs and diseases in a large-scale manner. Many methods have been developed in this direction, including: (i) matching drug indications by their disease-specific response profiles based on the Connectivity Map (CMap) dataset [5] and (ii) predicting novel associations among drugs and diseases by the ‘Guilt and Association’ (GBA) approaches [6]. Every method has its pros and cons. CMap approach relies on the dynamic gene expression datasets generated under different conditions and suffers from low precision [5]. GBA [6] approach takes advantage of disease associations with the same drug, but it is only applicable in the case that some indications for the drug in question are already known and complete.

Integrative analysis is one way out [7]. Recently, a novel integrative method was proposed for drug-disease association prediction [8]. This method heuristically summarized multiple drug-drug and disease-disease similarity measures from various aspects and repositioning was done based on the observation that similar drugs tend to treat similar diseases. The authors reported high specificity and sensitivity (AUC = 0.9). This approach applied logistic regression to integrate multiple drug-drug and disease-disease similarity metrics to collect the evidence for a strong association. This scheme provides a machine learning framework, and there is still much room to improve both from more general data collecting and accurate predicting.

In this paper, we construct a universal Predictor for Drug Repositioning (PreDR) to dissect drug-disease associations in a large-scale manner. We notice the rapid development of high-throughput technologies and ever-increasing accumulation of genome-wide datasets. On one hand, high-quality drug-disease networks have been constructed as the gold-standard to learn. On the other hand, drug’s functional roles in cell can be depicted from different aspects. For example, drug’s chemical structure provides information by the ‘structure determines function’ paradigm. Target protein provides the direct effect at molecular level, and side effect hints the unwanted effect at phenotype level. One straightforward idea is to learn understandable rules from these existing data and to predict novel drug-disease relationships. We demonstrate that drugs with similar chemical structures, target proteins, or side-effects will indicate similar diseases. Then we integrate heterogeneous chemical structures, target proteins, and side-effects information sources. Specifically, drug and disease are characterized by their similarity-based profiles, and kernel function is then defined to correlate drug with disease. Finally the potential drug-disease interactions are inferred by a machine learning model, i.e., support vector machine (SVM), which is motivated by statistical learning theory [9], [10] and has been proven successful on many different classification problems in bioinformatics [11]. PreDR provides an efficient way to overcome the main difficulty that these data sources are from three different levels and are extremely heterogenous.

PreDR is validated on a well-established drug-disease network with 1,933 interactions between 593 drugs and 313 diseases. By cross-validation, we find that all chemical structures, drug targets, and side-effects are predictive in different power. Combining these heterogenous properties predicts more drug-disease associations supported by literature and disease pathway database. Moreover, some novel predictions are supported by clinical trials database.

## Materials and Methods

**Figure 1:**
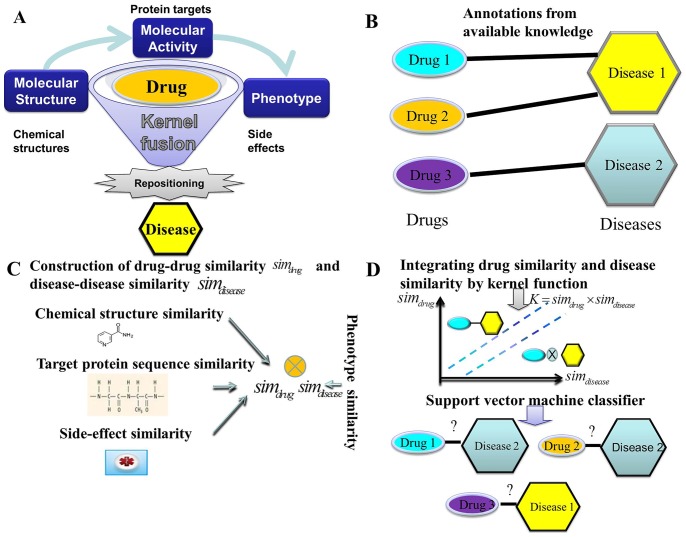
The summary of our method: PreDR. Subfigure A: The schematic plot for the PreDR method. Subfigure B: Collecting known interactions between drugs and diseases as gold standard positives in a bipartite graph. Subfigure C: Calculating the drug-drug and disease-disease similarity metrics. Subfigure D: Relating the similarity among drugs and similarity among diseases by kernel function, and applying SVM-based algorithm to predict unknown relationships among drugs and diseases.

We design a novel algorithm, named PreDR, to predict drug repositioning by associating known drugs with potential disease labels based on kernel fusion of heterogenous data sources. The schematic illustration of PreDR is shown in Figure 1A. The functional role of drug is characterized by its molecular structure, molecular activity, and phenotype data. PreDR aims to optimally integrate these three data sources and to connect drug with disease more accurately.

We treat drug-disease interaction prediction as a binary classification problem, i.e., determining whether a give pair of drug and disease is associated or not. We introduce SVM-based algorithm to cope with this task. The algorithm works in three phases (Figure 1): (Phase I) Collecting known drug-disease interactions as gold-standard positives in a bipartite graph. (Phase II) Modeling drug-drug and disease-disease similarity metrics. Drug similarity is derived from chemical structure, target protein, and side-effects. Disease similarity is based on semantic similarity of disease phenotypes [12]. (Phase III) Fusing the similarity among drugs and similarity among diseases by kernel methods, and applying SVM algorithm to predict the unknown relationships between drugs and diseases.

Given two drug-disease pairs, we consider to construct a kernel function which potentially correlates with their similarity. Since the kernel function represents the similarities among the training samples in some sense [13], we focus on the similarities among drugs and similarities among diseases. Therefore, we try to construct the similarity profile to represent drug and disease, respectively, in the following subsections.

### Collecting Structure, Activity, and Phenotype Data for Drugs

#### Chemical structure data

It is generally believed that drugs with similar chemical structure would carry out common therapeutic function, thus likely treat common diseases. So here, drugs are firstly represented by its chemical structure similarity profile.

PubChem database (http://pubchem.ncbi.nlm.nih.gov/) has defined 881 chemical substructures based on fingerprint search. Then a given drug can be represented by an 881 dimensional binary vector *x*. Each element of *x* is encoded as 1 or 0, which means the presence or absence of corresponding PubChem substructure. The description of these 881 chemical substructures is available at PubChem’s website. There are 107,292 associations between drugs and chemical substructures in the dataset, and each drug has 120.8 substructures on average [14]. The similarity between two drugs *d* and 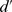 is evaluated by the weighted cosine correlation coefficient [15] as follows
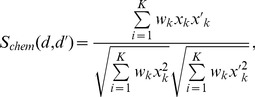
(1)where 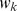 is the weight function for the k-th substructure, and defined as
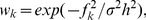
(2)where 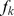 is the frequency of the k-th substructure, and 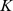 is the total number of substructures, 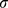 is the standard derivation of 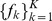, and 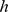 is a parameter and set to 10 in this study. The weight function puts more emphasis on rare substructures rather than frequent ones across different drugs, because rare substructures are more informative than common ones for specific function.

Suppose that we have 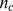 drugs in total, a matrix 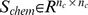 is then constructed to represent the chemical structure similarity matrix. Each row (or column) of this matrix is the chemical structure similarity profile for a single drug.

#### Drug-target interaction data

Drugs sharing common targets often possess similar therapeutic function. So there are many drug-target prediction studies for drug function. In our case, drugs interacting with the same targets are assumed to treat common diseases.

In this subsection, we represent drug-drug similarity by their target protein similarity. The high-quality drug-target interactions can be manually constructed from the KEGG BRITE [16], BRENDA [17], SuperTarget [18], and DrugBank [19]. In addition, the drug target interactions are well-studied for some specific protein families in previous studies [15], [20]–[23]. Here, we mainly collected drug-targets data from DrugBank [19], and defined drug similarity by target proteins’ sequence similarity. That is, given two drugs 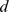 and 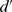, the similarity among them can be calculated as follows
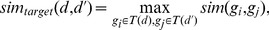
(3)where 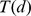 and 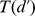 are the sets of target proteins, 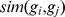 is sequence similarity among protein 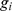 and 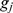, which is calculated by a normalized version of Smith-Waterman scores [20], [24].

The matrix 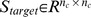 is then constructed to represent the compound target similarity matrix. Each row (or column) of this matrix is the target protein similarity profile for a single drug. Unlike chemical structure similarity matrix 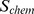, target protein similarity matrix 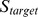 may not be a positive semidefinite matrix and needs the following normalization step
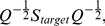
(4)where 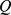 is diagonal matrix. The k-th diagonal element of 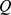 is 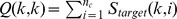.

#### Side-effect data

Drug side-effects, or adverse drug reactions, is one of the main causes of drug development failure and drug withdrawal from the market [14]. This high level phenotype data for drugs indicates the malfunction by off-targets. Thus side-effects data is useful to infer whether two drugs share similar target proteins [25]. In this study, drug side-effects are utilized to drug repositioning as some previous studies did [26], [27]. Similar to structure and target data, drug side-effects information is also applied to construct the drug similarity profile.

There are a total of 1,450 side-effect annotations in the SIDER database (http://sideeffects.embl.de/) for 888 approved drugs. Then each drug can be represented as an 1,450 dimensional binary vector 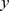. Each element of 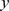 is encoded as 1 or 0 to indicate the presence or absence of corresponding side-effect. Drugs similarity under their side-effects metric is assessed by the weighted cosine correlation coefficient between 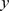 and 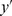 as follows
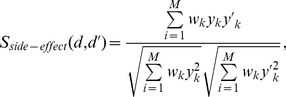
(5)where 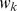 is the weight function for the k-th side-effect
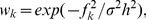
(6)where 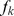 is the frequency of the k-th side-effect in the data, and 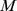 is the total number of side-effect, 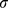 is the standard derivation of 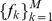, and 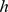 is a parameter (set to be 10 in this study).

The matrix 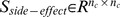 then represents the drug similarity matrix by their side-effects similarity. Each row (or column) of this matrix is the side-effect similarity profile for a single drug.

### Characterizing Disease Similarity by Phenotype Data

Similar to drug similarity profile, we used the disease similarity profiles to represent diseases. The disease-disease similarities were measured by their semantic similarity of disease phenotypes [12]. Text mining techniques were utilized to classify over 5,000 human phenotypes contained in the Online Mendelian Inheritance in Man (OMIM) database [12]. The phenotype similarity data are accessible through website at http://www.cmbi.ru.nl/MimMiner/. As a result, the similarity between two diseases 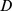 and 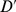 can be calculated as follows
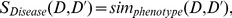
(7)where 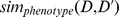 is semantic similarity of disease phenotype 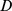 and 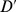, which is calculated by text mining approach in [12].

The matrix 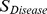 represents the similarities for all pairwise diseases. Each row (or column) is the phenotype similarity profile for a single disease.

### Kernel Fusion

With the representation of drugs and diseases by their similarity profiles, the similarity between two drug-disease pairs 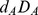 and 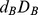 can be calculated as Kronecker product kernel [28]–[31] as follows
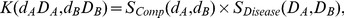
(8)where 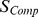 can be any one of 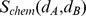, 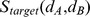, and 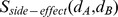 or their combination.

In this paper, “Chem” denotes the case when 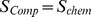, “Inter” denotes the case when 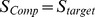, “Side-effect” denotes the case when 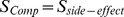, and “Comb” denotes the case when 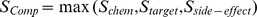, which means drug similarity supported by one or more than one metrics. Taken together, the rationale behind our kernel function construction scheme for drug-disease pairs is that two drug-disease pairs are similar only when the corresponding diseases and drugs are simultaneously similar supported by heterogeneous data sources.

### SVM Prediction with the Defined Kernel Function

With the above kernel function construction scheme, the drug-disease interactions prediction task is formalized as a binary classification problem. We treat the known drug-disease pairs as the gold-standard positives and the others as the gold-standard negatives. We note that this may cause the training data imbalance problem. Because there are more negatives and only a relatively small number of positives. This situation makes the SVM ineffective in determining the class boundary [32]. To maintain a balance, we randomly select a set of training negatives from the unlabelled data to have the same size with the training positives.

Feeding the kernel function in Equation (8) and training dataset to SVM, the classifier can be calculated by SVM algorithm.

### Benchmark Datasets and Algorithm Implementation

The benchmark dataset, which is used to test the performance of PreDR as a community standard, was summarized in [8]. It spans 1,933 associations between 593 drugs taken from DrugBank [19] and 313 diseases in OMIM database [33]. The drug chemical structure representation matrix was from [14] (http://cbio.ensmp.fr/yyamanishi/side-effect/), which contains 888 approved drugs represented by 881 substructures derived from PubChem [34]. Drug targets and targets sequences are from DrugBank [19]. The Smith-Waterman scores among protein sequences were calculated by MATLAB’s Bioinformatics Toolbox. Drug side-effects are from SIDER [35]. The disease phenotype similarity data was obtained at http://www.cmbi.ru.nl/MimMiner/.

We trained the SVM-based predictor by using 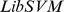 [36]. In our implementation, the penalty parameter 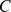 was optimized by a grid search approach with 3-fold cross-validation, and the optimal value of 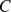 is 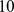. To evaluate the performance of our methods, 10-fold cross-validation was utilized. The performance of PreDR is shown by receiver operating characteristic (ROC) curve [37], which shows the trade-off between the true positive (correctly predicted interactions) rate (TPR) with respect to the false positive (wrongly predicted interactions) rate (FPR). Furthermore, the evaluation criteria shown in Table S1 are also applied to assess the performance rigorously.

## Results

### Chemical Structure, Drug-target Interactions, and Side-effects are all Predictive

We collect three data sources from structure, activity, and phenotype levels to characterize drugs: chemical structures, target proteins, and side-effects. First we test the fact that drugs with similar structures (target proteins or side-effects) will treat similar diseases. To show this, we correlate drug’s profile by chemical structure, target protein, side-effect similarity, and their curing disease profile. The drug similarity by disease profiling is defined as follows
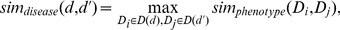
(9)where 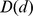 and 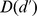 are the sets of diseases associated with drug 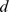 and 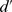 in gold standard positives, and 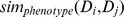 is the disease phenotype similarity calculated by text mining approach [12].

**Figure 2:**
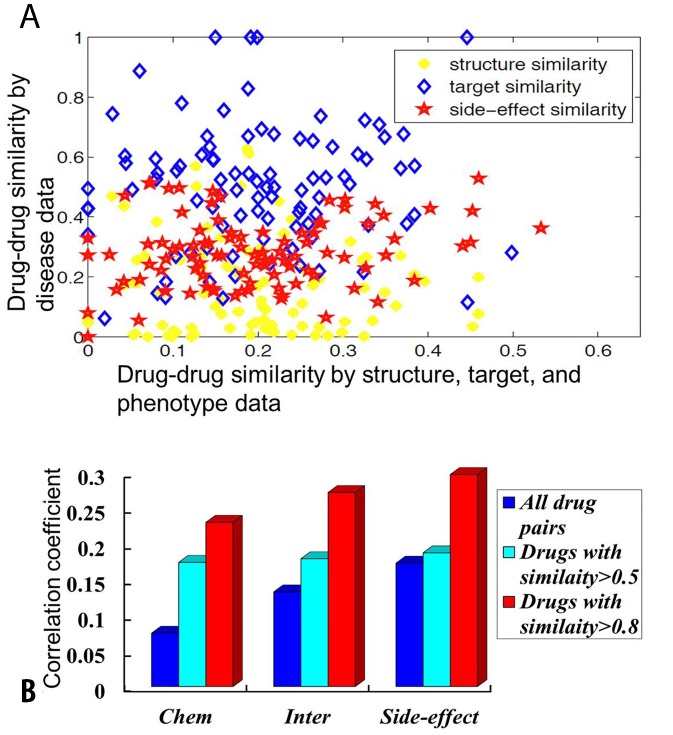
The relationship analysis between drug disease similarity profile and drug molecular structure, activity, and phenotype similarity profiles. Subfigure A: Scatter plot relating drug structures (yellow circles), targets (blue diamonds), side-effects (red stars) similarity with disease profile similarity. It shows that drug disease profile similarity is better correlated with its side-effect similarity, that is, drugs with similar side-effects similarity tends to cure similar diseases. Subfigure B: Barplot of the PCCs between structures, targets, side-effects similarity and disease profile similarity. All the p-values are smaller than 1e-2.

Figure 2A plots the weak correlations between drug similarity by their structures, protein targets, side-effects with drug similarity by its disease profile. It shows that drug’s disease profile similarity is more correlated with its side-effect similarity comparing with chemical structure and protein targets similarity. The Pearson’s correlation coefficients (PCCs) between drug’ disease profile similarity and the similarity from chemical structures, target proteins, and side-effects data are shown in Figure 2B. It shows that the correlation coefficients tend to be larger when two drugs are more similar. For example, the correlation coefficients are all larger than 0.2 with high confidence when drugs are similar than 0.8 for all three kind of data sources. Correlation coefficient between side-effects profile based similarity and disease profile based similarity is larger than 0.3 (Figure 2B). Taken together, chemical structure, target protein, and side-effect similarity correlate with drug’s disease profile similarity, i.e., drugs similar in either structure, target, or side-effects tend to cure similar diseases.

In addition to global similarity by disease profile, we also correlate the similarity obtained from three kinds of data sources with the drugs’ distance in the known drug-disease interaction network. We define the distance of two drugs in the network as the length of the shortest path between them in the network. We plot the distributions of chemical structure, target protein, and side-effects similarity scores with respect to network distance in Figure S1. It shows that all three kinds of similarities are larger than 0.6 for 75% drug pairs sharing common diseases. That is, two drugs with larger similarity scores in the three data sources tend to share common diseases.

All the facts suggest predictability of different data sources for drug-disease associations. This analysis provides support for our follow-up integrative analysis.

### Drug Repositioning by Single Data Source

In this subsection, we assess the effects of chemical structures, target proteins, and side-effects in drug repositioning prediction. Their performances are evaluated and visualized by ROC curves [37].

**Figure 3:**
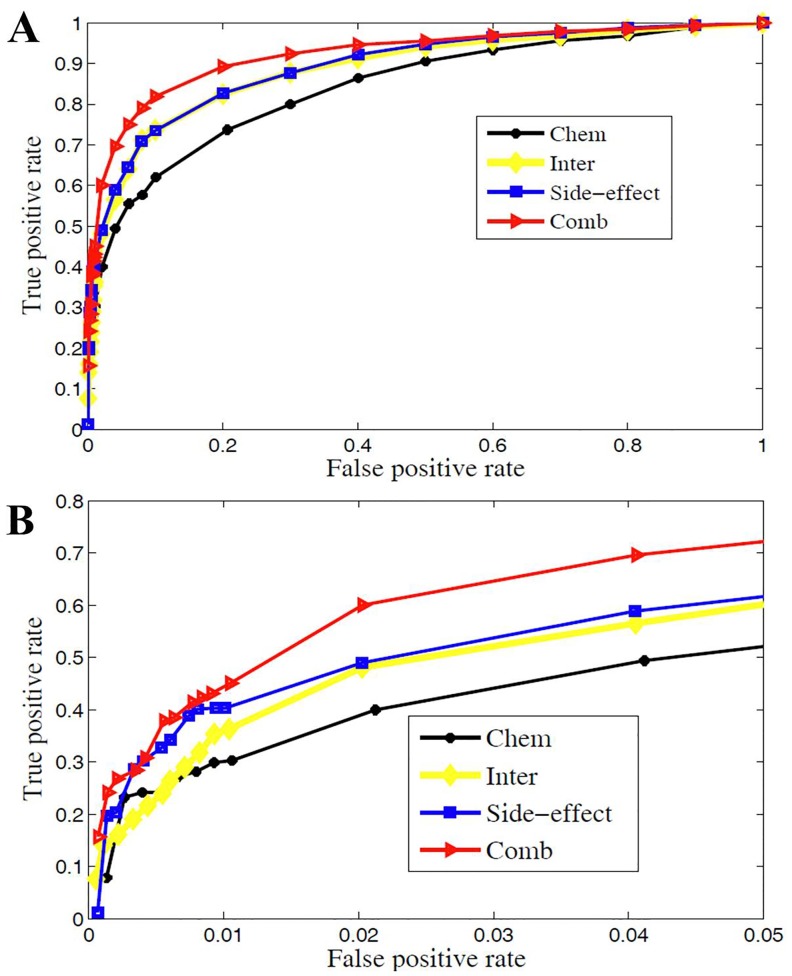
The performance of predictions are shown as ROC curves. Subfigure A: The ROC curves for three data sources (“Chem”: chemical structure, “Inter”: target protein, “Side-effect”: side-effect based similarity and “Comb”: integration of “Chem”, “Inter”, and “Side-effect”). “Side-effect” is general more predictive for more experimentally observed drug-disease associations. Subfigure B shows the ROC curves with false positive rate (FPR) less than 0.05. “Chem” obtains the highest true positive rate (TPR) when FPR is very small.

Firstly, we replace the drug similarity matrix 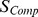 in kernel function (8) (see Materials and Methods) with 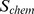, 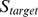, and 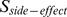 to test the effect of chemical structure, target protein, and side-effects similarity in uncovering the experimentally observed drug-disease interactions. The ROCs for each data source are displayed in Figure 3A. It shows that all the ROC curves are beyond the diagonal (random classification) and closer to the 0–1 baseline. The corresponding evaluation criteria when the corresponding F-measure reaches its maximum are listed in Table 1. We can see that “Chem” obtains AUC 0.83 and Sn 0.83. That is, chemical structure is useful in drug-disease interaction prediction. Target proteins and side-effects play their important roles in predicting drug-disease interactions too. The Accs, Sns, Pres and F-measures are all larger than 0.8 for “Inter” and “Side-effect”, and AUCs reach 0.88. It indicates that target proteins and side-effects can address the activity and effect of drug in cell thus uncover more experimentally observed drug-disease interactions.

**Table 1:** The performance comparison for different data sources.

Data source	AUC	Acc	Sn	Sp	Pre	F-measure
Chem	0.834	0.763	0.737	0.792	0.781	0.763
Inter	0.889	0.812	0.824	0.799	0.804	0.811
Side-effect	0.894	0.813	0.826	0.799	0.804	0.812
**Comb**	**0.902**	**0.823**	**0.847**	**0.799**	**0.808**	**0.822**

The best predictions are highlighted in bold.

Since we are more interested in the performance of these methods when FPR is rather small, we also draw ROC curves when FPR is less than 0.05 in Figure 3B. It shows that, “Chem” obtains the highest TPR when FPR is very small, and with the number of known interactions increasing, “Side-effect” reveals more experimentally observed drug-disease interactions. All these results suggest that, each data source will do one’s bit in prediction. Therefore, combination of these three data sources produces a much more sophisticated picture of the interactions among drugs and diseases.

### Data Fusion Improves Drug Repositioning

The usefulness of each data source is validated in uncovering the experimentally observed indications for drugs. In the following, we validate the effect of combination of three data sources.

The performances of combination method: “Comb” is also evaluated and visualized by ROC curve in Figure 3 and various evaluation criteria in Table 1. Figure 3 shows that, “Comb” not only obtains the best area under ROC curve, but also achieves the highest TPR when FPR less than 0.05. This specifically demonstrates that “Comb” improves performance when predicting a small fraction of known drug-disease interactions as positives.

Table 1 shows that, “Comb” performs better than using single data source. For example, “Inter” and “Side-effect” reach the AUC 0.889 and 0.894, respectively, while “Comb” obtains an AUC 0.902. “Inter” and “Side-effect” obtain F-measures 0.811 and 0.812, respectively. “Comb” obtains a F-measure 0.822 and improves by one percent. These facts demonstrate that the significant improvement is obtained by data integration.

For drug-disease interaction prediction task, the gold standard positives are relatively not abundant. The area under precision-recall curve [38] (AUPR) is a more significant quality measure than the AUC, as it punishes much more the existence of false positive examples found among the best ranked prediction scores [39]. So we use the AUPRs (Figure S2) and precision-recall curves (Figure S3) to validate our results. All results shown in Figure S2 and Figure S3 suggest that each data source is predictive and data integration brings the improvement.

### Leave One Drug Out Cross-validation

**Figure 4:**
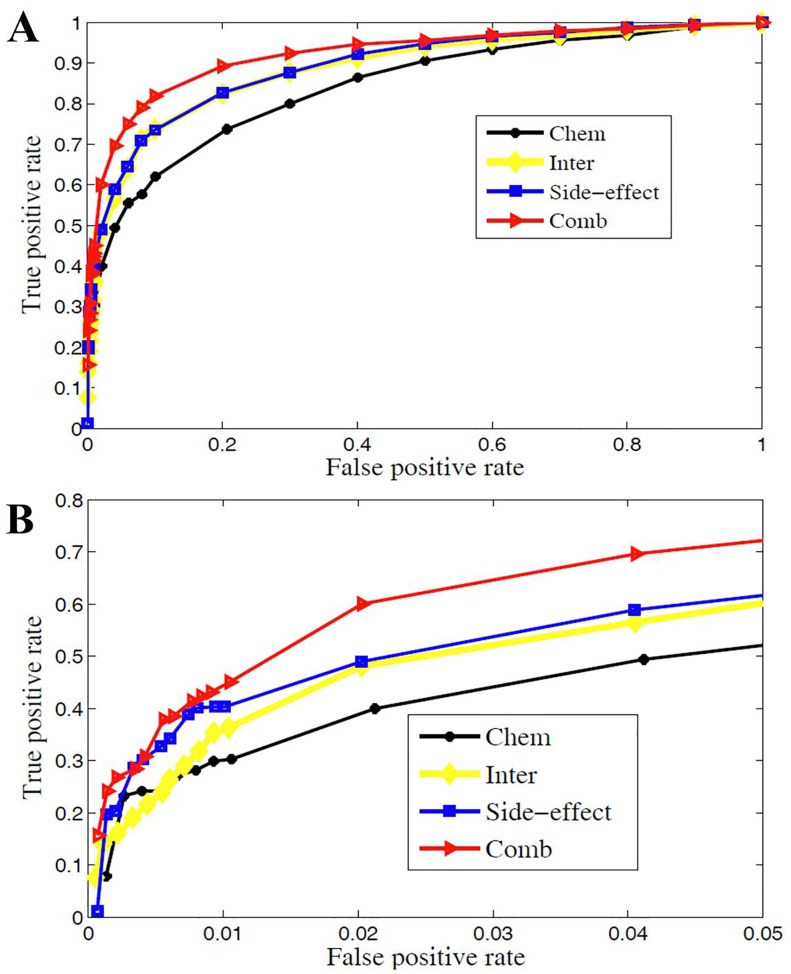
Leave one drug out cross-validation. Subfigure A: The procedure for leave one drug out cross-validation. Subfigure B: The AUCs obtained from leave one drug out cross-validation (“Chem”: chemical structure, “Inter”: target protein, “Side-effect”: side-effect, and “Comb”: integration of “Chem”, “Inter”, and “Side-effect”). It further shows that all three data sources can uncover new diseases for a novel drug, and integration works even better.

Given a new drug, people are interested in which disease it will cure, i.e., whether this novel drug is related with known diseases. To this end, we test the performance of our method by doing leave one drug out cross-validation. That is, we exclude each drug and its interactions from gold standard positives (known drug-disease interaction network). This drug and its interactions are taken in turn as a test dataset to validate the model trained on the remaining drug-disease interaction network. The procedure is illustrated in Figure 4A. The AUCs for leave one drug out cross-validation are shown as barplot in Figure 4B. The results are similar to 10 fold cross-validation results. “Chem” achieves the worst AUC, “Inter” obtains a better one, and “Side-effect” performs the best. Furthermore, all three data sources have larger AUCs than 0.78. “Inter” and “Side-effect” make AUC 0.80 and 0.84, respectively. “Comb” receives an AUC 0.85. These results demonstrate the data source complementarily and utility of heterogeneous data integration.

### Comparing with Previous Work

**Figure 5:**
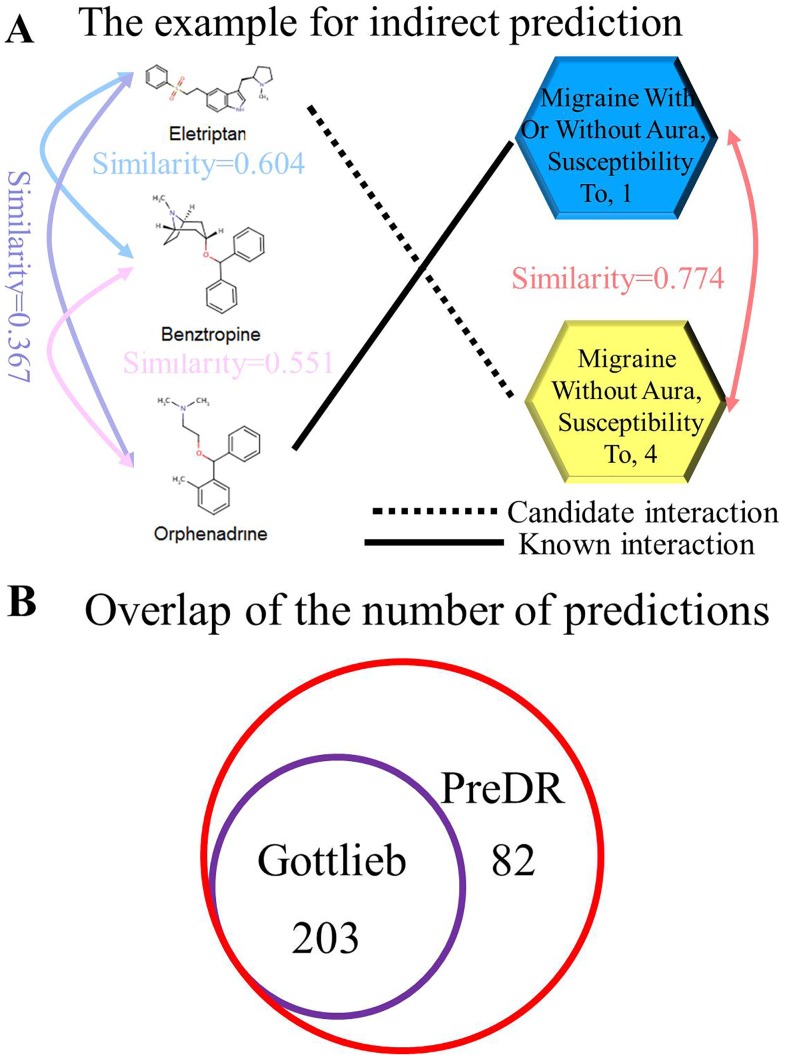
Comparison with previous method. Subfigure A: An example for ‘indirect drug-disease association’. The candidate drug disease association is revealed utilizing the drug ‘Benztropine’ as a bridge to connect drug ‘Eletriptan’ and drug ‘Orphenadrine’. Subfigure B: The overlap of predictions by our method PreDR and previous method.

We compared PreDR with previous work in [8] since the gold-standard positives used in our study is the same. The authors in [8] measured the similarity of the pertaining drug and disease only for the nearest known associated drug-disease pair. Specifically, a simple geometric mean based score is calculated to combine the drug-drug similarity with disease-disease similarity, and the maximal score with the known associated drug-disease pair is extracted as classification feature [8]. Differently, we measured similarities among all the drugs and among all the diseases to represent drug and disease, respectively. And then we use kernel function and SVM classifier to train the model. That is, we utilize the global information extracted from drug-disease data in PreDR. To show this advantage, we illustrated one example in Figure 5A. Here the candidate association between drug and disease (shown as black dash line in Figure 5A) cannot be inferred directly by the most similar known association (shown as black solid line in Figure 5A). Because drug ‘Eletriptan’ is not very similar to drug ‘Orphenadrine’ (similarity score is 0.367). However our method can utilize the drug ‘Benztropine’ as a bridge to connect drug ‘Eletriptan’ and drug ‘Orphenadrine’. In this way we can have more confidence to associate candidate drug-disease pair. Because this prediction is achieved by the indirect drug similarity and we call it as ‘indirect drug-disease association’. Indeed, more drug-disease associations can be uncovered by PreDR (Figure 5B).

On cross-validation accuracy, the authors in [8] had obtained an AUC 0.9 in predicting drug indications. In our study, “Comb” achieves an AUC 0.91, which is nearly the same as the authors obtained in [8]. The authors in [8] used more data sources to measure the drugs and diseases associations, including GO annotations for target proteins, the genetic based disease similarity from gene expression experiments and so on. Given the fact that we use less data sources, PreDR works well to achieve comparable performance. We note that these data sources can be easily integrated into PreDR. Since our aim here is to demonstrate a useful data integrative analysis framework instead of the most comprehensive and accurate predictions. We only pick one representative data source from the structure, activity, and phenotype levels. Thus we have the sufficient reason to believe that the improvement can be expected by introducing more data for each level.

### Novel Predictions

**Figure 6:**
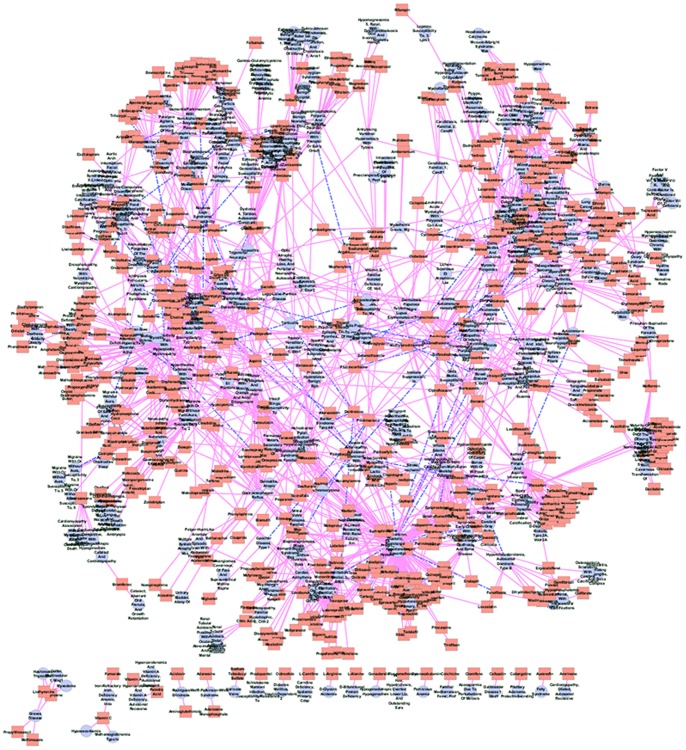
The predicted drug-disease network (only top 100 novel predictions are shown). LightCoral rectangle represents drug and LightSteelBlue cycle represents disease. Pink solid line represents the known interaction and the DarkBlue dash line represents the new prediction.

**Figure 7:**
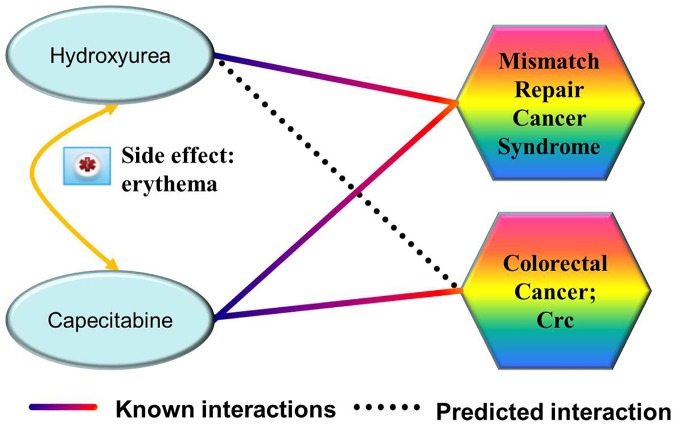
The most confident prediction achieved by PreDR. Disease ‘Colorectal Cancer; Crc’ is revealed because that the similar drug ‘Capecitabine’ which shares the same side-effect ‘erythema’, treats disease ‘Colorectal Cancer; Crc’.

**Figure 8:**
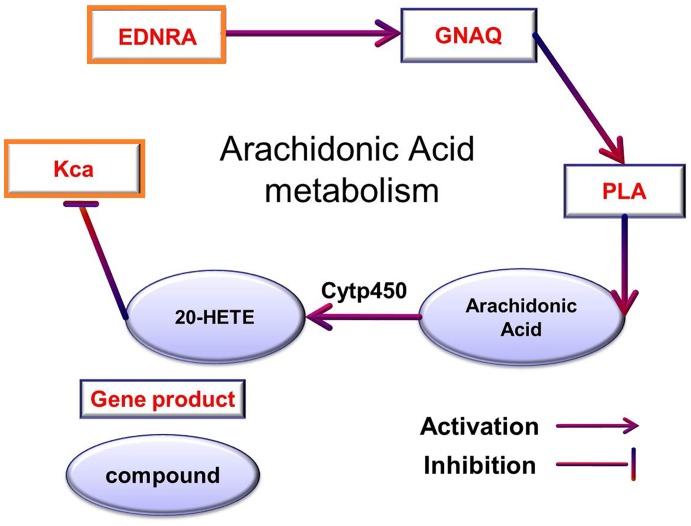
Pathway ‘Arachidonic Acid metabolism’. Drug target proteins and disease genes are highlighted by orange border.

In this subsection, we test whether PreDR can produce biologically useful predictions. To this end, we focus on the unknown (non-interacting) drug-disease pairs. We used kernel “Comb” on the gold standard positives and randomly selected gold standard negatives from the unlabelled pairs, and tested it on the remaining drug-disease pairs. Our expectation is that “Comb” can discover many missing associations. We drew the predicted drug-disease network in Figure 6 (only top 100 newly predicted interactions are shown for conciseness). Take drug ‘Hydroxyurea’ as an example, disease ‘Colorectal Cancer; Crc’ is revealed because that the similar drug ‘Capecitabine’ which shares the same side-effect ‘erythema’, treats disease ‘Colorectal Cancer; Crc’ (illustrated in Figure 7). The top five novel predictions are listed in Table S2. For each novel prediction, we checked the drug target proteins form DrugBank [19], the disease genes from OMIM [33], and the corresponding pathway information from KEGG BRITE [16]. Finally, we checked whether novel predictions appear in current clinical trials database (http://clinicaltrials.gov/). Take the most confident prediction as an example, target protein ‘Endothelin-1 receptor’ (EDNRA) for ‘Bosentan’, and the disease gene ‘KCNMB1’ (Kca) for ‘Hypertension, Diastolic, Resistance To’ belong to the same pathway ‘Arachidonic Acid metabolism’ (Figure 8). Furthermore, we find that this drug-disease pair appears in current clinical trials, the ‘ClinicalTrials.gov Identifier’ is NCT00820352. That is, this novel drug-disease association may be true with high probability.

The target of ‘Hydroxyurea’ is ‘Ribonucleoside-diphosphate reductase large subunit’ (RRM2: p53R2), the disease gene of ‘Colorectal Cancer; Crc’ is ‘TP53’ (p53). In addition EDNRA and Kca belong to the same pathway hsa04115 (shown in Figure 8). Unfortunately, we do not find the evidence of this pair in current clinical trials database. However, the lower pathway distance between disease gene and target already shows the high probability for their association.

The target of ‘Dasatinib’ is ‘Tyrosine-protein kinase ABL1’ (ABL1: BCRABL), the disease gene of ‘Leukemia, Acute Myeloid; Aml’ is ‘Mast/stem cell growth factor receptor Kit’ (KIT). In addition ABL1 and KIT belong to the same pathway hsa05200. Furthermore. this drug disease pair is found the current clinical trials database, the ‘ClinicalTrials.gov Identifier’ are NCT01392703 and NCT00850382. It means that this novel pair may interact in vivo with high probability.

The target of ‘Nabumetone’ is ‘Prostaglandin G/H synthase 2’ (PTGS2), the disease gene of ‘Sensory Ataxic Neuropathy, Dysarthria, And Ophthalmoparesis; Sando’ is ‘DNA polymerase subunit gamma-1’ (POLG). In addition ABL1 and KIT belong to a same pathway hsa01100. No evidence is found for this pair in clinical trials database.

The target of ‘Acebutolol’ is ‘Beta-1 adrenergic receptor’ (ADRB1: ADR), the disease gene of ‘Alcohol Dependence’ is ‘Gammaaminobutyric acid receptor subunit alpha-2’ (GABRA2: GABR). In addition ABL1 and KIT belong to a same pathway hsa04080 No evidence is found for this pair in current clinical trials database. The lower pathway distance between disease gene and target shows the high probability for their association.

Among our top five predictions, two of them are supported by current clinical trials. All these results suggest that, PreDR can uncover potential repositioning of drugs, and can provide candidates for further high-resolution validation.

## Discussion

In this paper, we propose a new computational method, PreDR, to predict drug repositioning. PreDR allows us to infer novel associations among drugs and diseases by integrating heterogeneous data sources. Our main contributions here are both in integrating the heterogeneous drug and disease similarity profiles by kernel function and construction of a predictive model.

Specifically, we characterize the drug similarity profiles form three levels. Chemical structures, target proteins, and side-effects data are collected to represent structure, activity, and phenotype for drugs. Treating the task as a binary classification problem, we train a SVM-based predictor to uncover unknown interactions between drugs and diseases. The improvement in various evaluation criteria is obtained on a well-established dataset with 1,933 interactions among 593 drugs and 313 diseases. Leave one drug out cross-validations, database search, literature survey, and functional annotation analysis reveal that PreDR provides high quality predictions. For example, among the top five novel predictions, two of them are supported by current clinical trials database. Taken together, PreDR can serve as a useful tool for drug repositioning and promote the further drug discovery.

One possible concern is that PreDR works well by those ‘trivial’ predictions. For example, those drugs sharing common target are easily to be predicted to cure the same diseases. To address this issue, we test our PreDR by filtering out the potential “trivial’ predictions. Take target protein as an example, we filter the target proteins with high sequence similarity (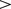
0.8). That is, the drugs with high sequence similarity targets (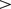
0.8) are excluded from gold standard positives. On this filtered dataset, we validate PreDR’s prediction performance. We achieve the AUC 0.754 for “Inter”, which is lower than 0.889 obtained by “Inter” on the full gold standard positive dataset, but much higher than 0.5 (random classification). This experiment suggests that PreDR can reveal ‘non-trivial’ predictions, by fully considering the global and remote similarity in kernel function.

In this article, we attempt to improve the performance by integrating target proteins information. The experimental results show that, comparing with chemical structures, the performance is indeed improved by characterizing drugs in target sequence-based similarity. In fact, there are other ways to define the drug similarity based on their targets. For example, target closeness in protein-protein interaction (PPI) network can be used [8]. Therefore we take the targets closeness in a human PPI network derived from HPRD (Release 9) to incorporate into PreDR. Unfortunately, the prediction accuracy is worse than sequence-based similarity. This may be due to the high false positive rate and relative low precision of single PPI network. In the future, we will define the targets closeness based on an integrated human PPI networks collected from multiple curated databases, including HPRD [40], OPHID [41], and BIND [42] databases.

For disease, we only apply the phenotypic similarity in current study. Studies have shown that phenotypically similar diseases are often caused by functionally related genes [43]. In addition, many large-scale studies support the idea that genes sharing similar diseases are tightly linked in the network [44], [45]. Therefore, disease genes closeness in a PPI network is useful to correlate disease with candidate genes [43]. Apart from gene closeness, genes with similar sequences may be functionally related [46], [47]. It is promising to use disease gene sequence similarity and closeness in a PPI network to characterize disease. So we applied disease gene sequence similarity and closeness in a human PPI networks to measure the disease similarity, and then extend PreDR. Unfortunately, neither sequence similarity nor closeness in a human PPI networks can achieve better results than phenotype-based similarity (see Table S3). The inefficient performance may be due to the fact that the gap between phenotype (disease) and genotype (gene) is too large and the associations are not so accurate. One possible way out is to validate the disease gene based similarity by GO annotation terms, which may closely correlate with the disease similarity.

## Supporting Information

Figure S1 **The distribution of drug similarity scores among the drugs sharing common diseases (Distance is 2 for Drug1 and Drug 2), mediate (Distance is 4 for Drug1 and Drug 2) or unrelated (Distance is 6 for Drug1 and Drug 2), respectively.** Figure S1 shows that the drugs sharing common disease tend to have higher side-effect similarity comparing with the structure and target protein similarity.
(TIF)

Figure S2 **The AUPRs derived from different similarity measurements (Chem: chemical structure, Inter: drug target interaction, Side-effect: side-effect based similarity and Comb: integration of Chem, Inter, and Side-effect).** Figure S2 shows that all chemical structures, target proteins, and side-effects are predictive in drug repositioning prediction, and improved performance can be achieved by integration of them.
(TIF)

Figure S3 **The precision-recall curves derived from different similarity measurements (chem: chemical structure, inter: drug target interaction, side-effect: side-effect based similarity, and comb: Integration of chem, inter, and side-effect).** Figure S3 presents, all methods make precision higher than 0.7 when recall value is larger than 0.8, and comb achieves the highest precision with higher recall values. All these results suggest that each data source is predictive and by combination further performance improvement can be obtained.
(TIF)

Table S1 **The definitions of evaluation criteria.** Table S1 lists the evaluation criteria used in this article. Here TP is the number of drug-disease pairs correctly predicted to interact, FP is the number of drug-disease pairs predicted to interact but actually not. And TN is the number of drug-disease pairs do not interact and predicted correctly, FN is the number of drug-disease pairs predicted not to interact but actually interact.
(PDF)

Table S2 **The top five drug repositioning predictions by our method.** Table S2 presents the top five novel predicted drug-disease interactions.
(PDF)

Table S3 **The performance comparison of disease gene closeness in a human PPI network under different drug similarity measurements to predict drug repositioning. The best predictions obtained are highlighted in bold.** Table S3 just lists the performance of disease gene closeness in PPI network due to the fact that disease gene sequence similarity performs worse than its closeness in PPI network.
(PDF)
