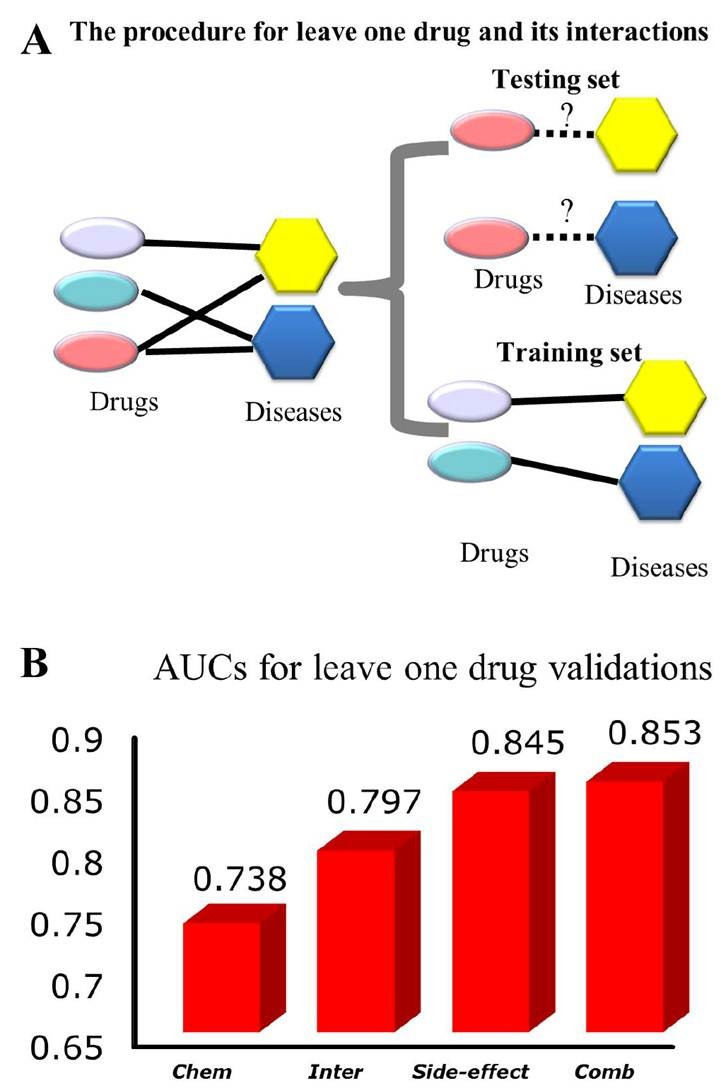# Correction: Drug Repositioning by Kernel-Based Integration of Molecular Structure, Molecular Activity, and Phenotype Data

**DOI:** 10.1371/annotation/fe02e998-6a38-4fd7-9df6-241bc4d0f267

**Published:** 2013-12-13

**Authors:** Yongcui Wang, Shilong Chen, Naiyang Deng, Yong Wang

Figure 3 erroneously appears in place of Figure 4. Please see the correct Figure 4 here: 

**Figure:**