# Supplementary figures and images for: Drug Repositioning by Kernel-Based Integration of Molecular Structure, Molecular Activity, and Phenotype Data
Source: PLoS One. 2013 Nov 11;8(11):e78518. doi: 10.1371/journal.pone.0078518 (PMC3823875; doi:10.1371/journal.pone.0078518)

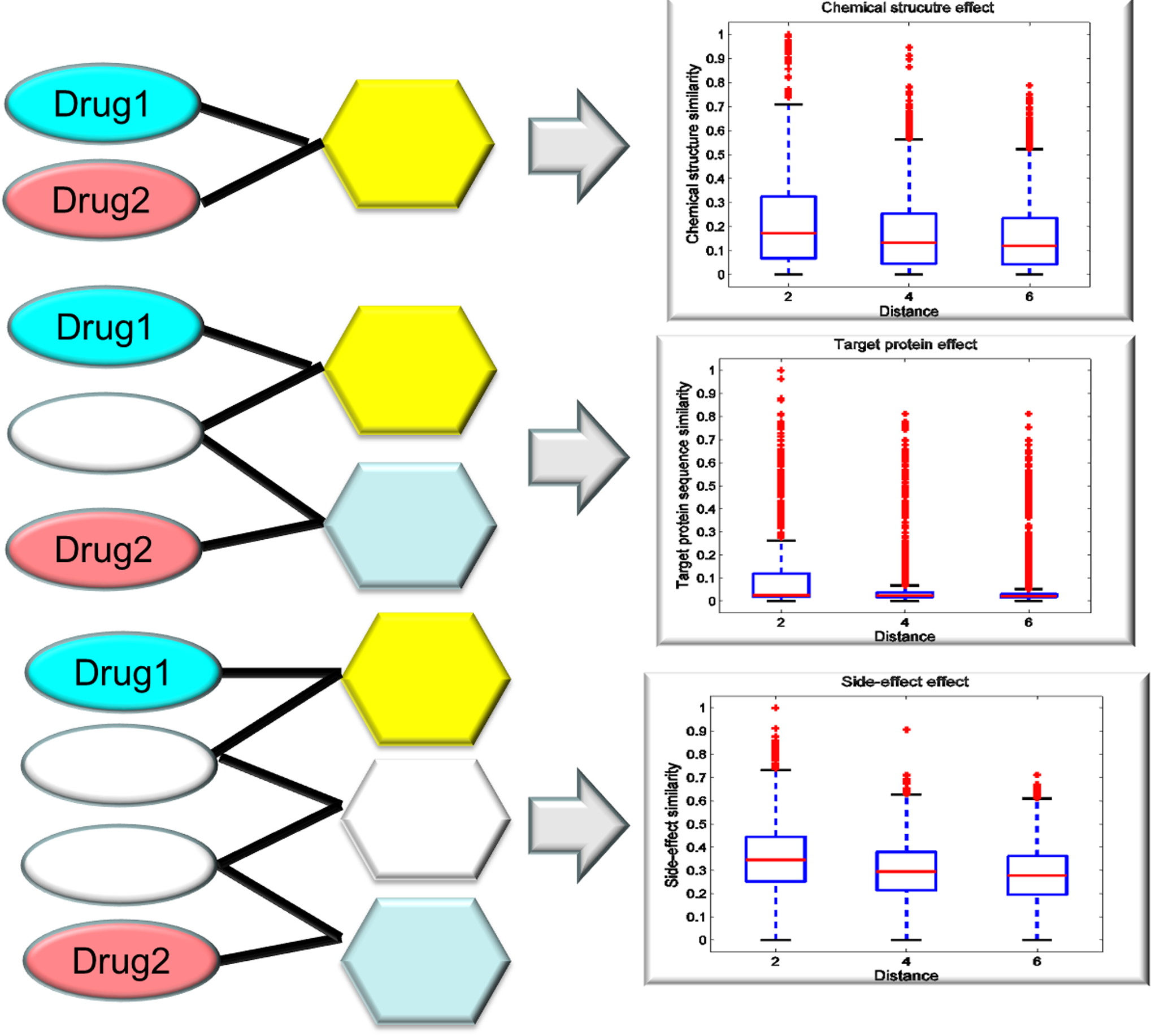

Supplement: Figure S1 — The distribution of drug similarity scores among the drugs sharing common diseases (Distance is 2 for Drug1 and Drug 2), mediate (Distance is 4 for Drug1 and Drug 2) or unrelated (Distance is 6 for Drug1 and Drug 2), respectively. Figure S1 shows that the drugs sharing common disease tend to have higher side-effect similarity comparing with the structure and target protein similarity. (TIF) [file pone.0078518.s001.tif]

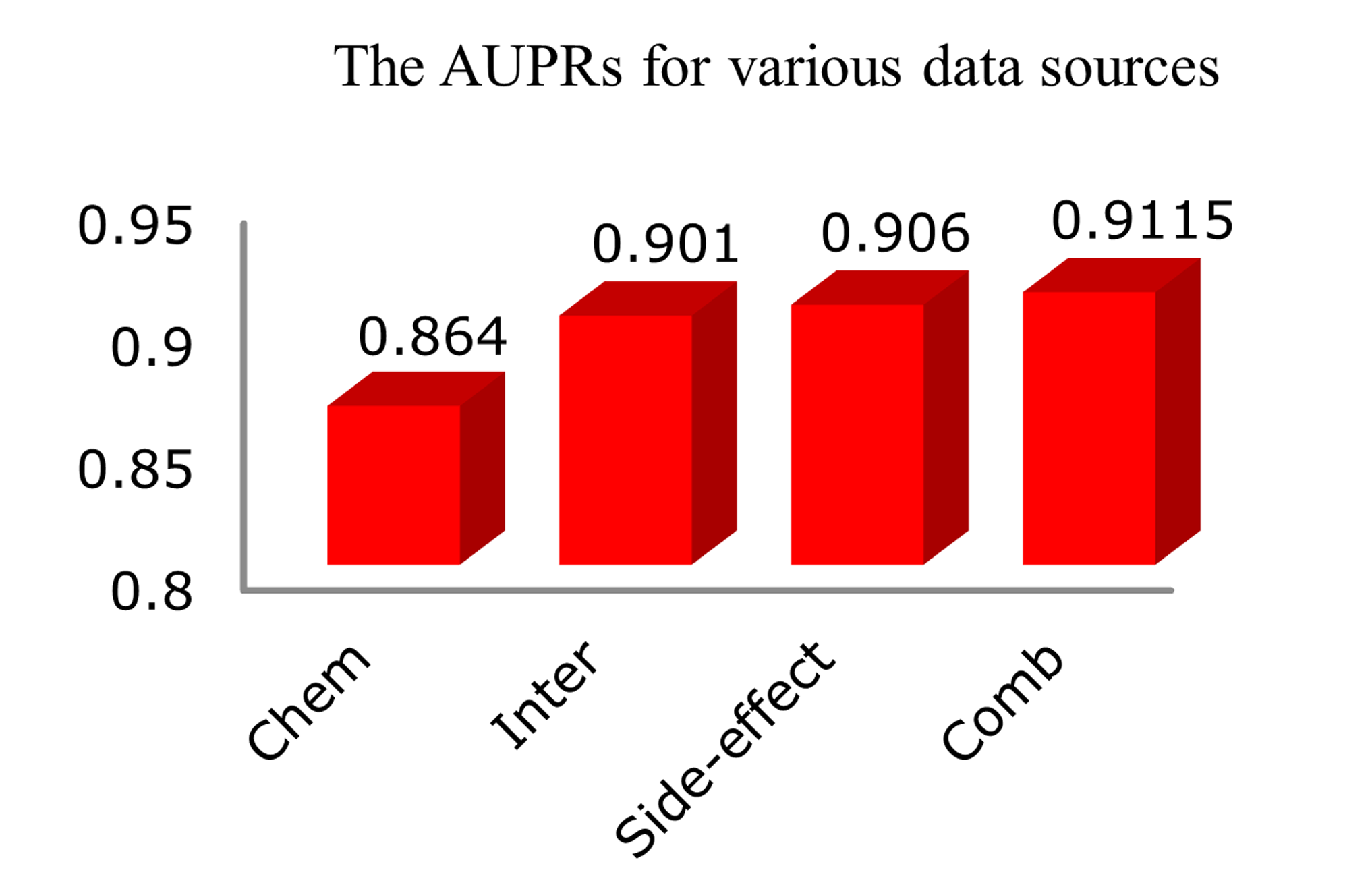

Supplement: Figure S2 — The AUPRs derived from different similarity measurements (Chem: chemical structure, Inter: drug target interaction, Side-effect: side-effect based similarity and Comb: integration of Chem, Inter, and Side-effect). Figure S2 shows that all chemical structures, target proteins, and side-effects are predictive in drug repositioning prediction, and improved performance can be achieved by integration of them. (TIF) [file pone.0078518.s002.tif]

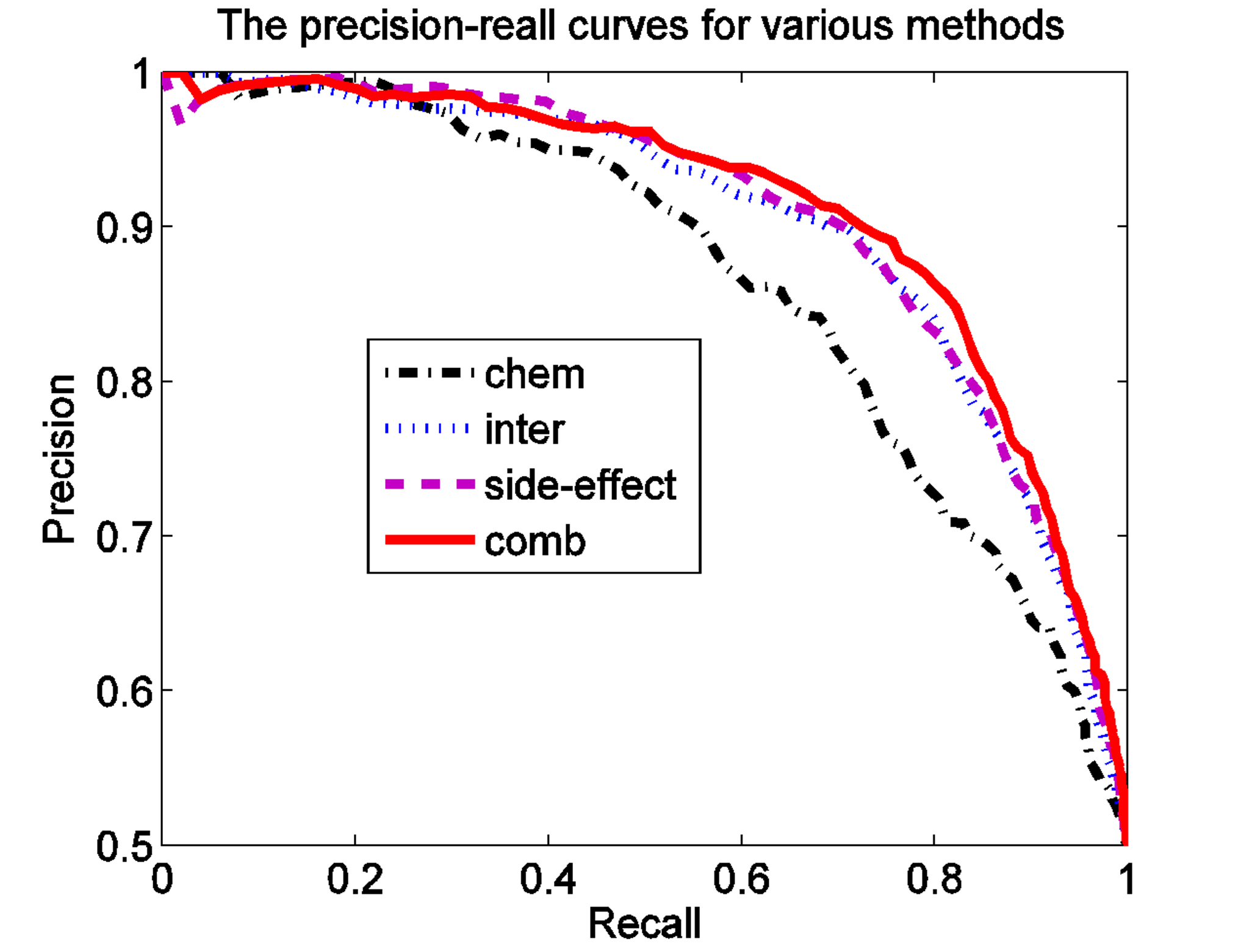

Supplement: Figure S3 — The precision-recall curves derived from different similarity measurements (chem: chemical structure, inter: drug target interaction, side-effect: side-effect based similarity, and comb: Integration of chem, inter, and side-effect). Figure S3 presents, all methods make precision higher than 0.7 when recall value is larger than 0.8, and comb achieves the highest precision with higher recall values. All these results suggest that each data source is predictive and by combination further performance improvement can be obtained. (TIF) [file pone.0078518.s003.tif]
