# Supplementary material for: Drug Repositioning by Kernel-Based Integration of Molecular Structure, Molecular Activity, and Phenotype Data
Source: PLoS One. 2013 Nov 11;8(11):e78518. doi: 10.1371/journal.pone.0078518 (PMC3823875; doi:10.1371/journal.pone.0078518)
Supplement: Table S1 — The definitions of evaluation criteria. Table S1 lists the evaluation criteria used in this article. Here TP is the number of drug-disease pairs correctly predicted to interact, FP is the number of drug-disease pairs predicted to interact but actually not. And TN is the number of drug-disease pairs do not interact and predicted correctly, FN is the number of drug-disease pairs predicted not to interact but actually interact. (PDF) [file pone.0078518.s004.pdf]

**Table S1**

| **Evaluation criterion** | **Description** |
|---|---|
| AUC | area under the ROC curve |
| Accuracy (Acc) | TP+TN/TP+TN+FP+FN |
| Sensitivity (Sn) | TP/TP+FN |
| Specificity (Sp) | TN/TN+FP |
| Precision (Pre) | TP/TP+FP |
| F-measure | 2×Sn×Sp/Sn+Sp |
