# Supplementary material for: Drug Repositioning by Kernel-Based Integration of Molecular Structure, Molecular Activity, and Phenotype Data
Source: PLoS One. 2013 Nov 11;8(11):e78518. doi: 10.1371/journal.pone.0078518 (PMC3823875; doi:10.1371/journal.pone.0078518)
Supplement: Table S2 — The top five drug repositioning predictions by our method. Table S2 presents the top five novel predicted drug-disease interactions. (PDF) [file pone.0078518.s005.pdf]

Table S2

**1st**	Drug	Bosentan
	Indication	Hypertension,
	Target	Endothelin-1
	Disease	Gene
	Pathway	hsa04270
**2nd**	Drug	Hydroxyurea
	Indication	Colorectal
	Target	Ribonucleoside-diphosphate
	Disease	Gene
	Pathway	hsa04115
**3rd**	Drug	Dasatinib
	Indication	Leukemia,
	Target	Tyrosine-protein
	Disease	Gene
	Pathway	hsa05200
**4th**	Drug	Nabumetone
	Indication	Sensory
	Target	Prostaglandin
	Disease	Gene
	Pathway	hsa01100
**5th**	Drug	Acebutolol
	Indication	Alcohol
	Target	Beta-1
	Disease	Gene
	Pathway	hsa04080
