# Supplementary material for: Drug Repositioning by Kernel-Based Integration of Molecular Structure, Molecular Activity, and Phenotype Data
Source: PLoS One. 2013 Nov 11;8(11):e78518. doi: 10.1371/journal.pone.0078518 (PMC3823875; doi:10.1371/journal.pone.0078518)
Supplement: Table S3 — The performance comparison of disease gene closeness in a human PPI network under different drug similarity measurements to predict drug repositioning. The best predictions obtained are highlighted in bold. Table S3 just lists the performance of disease gene closeness in PPI network due to the fact that disease gene sequence similarity performs worse than its closeness in PPI network. (PDF) [file pone.0078518.s006.pdf]

Table S3

Data source	AUC	Acc	Sn	Sp	Pre	F-mearsure
**Chem**	0.667	0.633	0.698	0.568	0.653	0.626
**Inter**	0.786	0.710	0.720	0.699	0.705	0.709
**Side-effect**	0.803	0.742	0.685	0.799	0.773	0.738
**Comb**	**0.811**	**0.749**	**0.699**	**0.799**	**0.777**	**0.75**
